# MolVE: An Open-Source Web Platform for Visualizing and Evaluating AI-Designed Molecules to Aid in Prioritization

**DOI:** 10.1021/acs.jcim.5c02412

**Published:** 2026-02-10

**Authors:** Davide Rigoni, Alessandro Sperduti, Stefano Moro

**Affiliations:** † Department of Mathematics “Tullio Levi-Civita”, University of Padova, Via Trieste 63, Padova 35131, Italy; ‡ Fondazione Bruno Kessler, Via Sommarive 18, Povo 38123, Italy; § Human Inspired Technology Research Centre, University of Padova, Via Luigi Luzzatti 4, Padova 35121, Italy; ∥ Department of Information Engineering and Computer Science, University of Trento, Via Sommarive 9, Trento 38123, Italy; ⊥ Department of Pharmaceutical and Pharmacological Sciences, 9308 University of Padova Via Marzolo 5, Padova 35131, Italy

## Abstract

Advances in artificial intelligence and deep generative models have enabled the rapid generation of novel molecular structures for advanced material science and drug discovery. However, the effective evaluation of these candidates still depends, in the end, on expert judgment, which is often fragmented and difficult to scale. This work introduces MolVE, an open-source, web-based platform designed to enable asynchronous, distributed, and collaborative evaluation of molecules by experts. The platform combines secure user authentication, data set management, and interactive 2D/3D visualizations, enabling chemists and pharmacologists to curate, annotate, and evaluate molecules efficiently. Additionally, it offers APIs for seamless integration with programming languages and provides a Python service to run machine learning and deep learning models. MolVE is a comprehensive, ready-to-deploy application built with React, Node.js, Express, and PostgreSQL, and is fully containerized using Docker. This setup enables scalable deployment in both academic and industrial settings.

## Graphical abstract

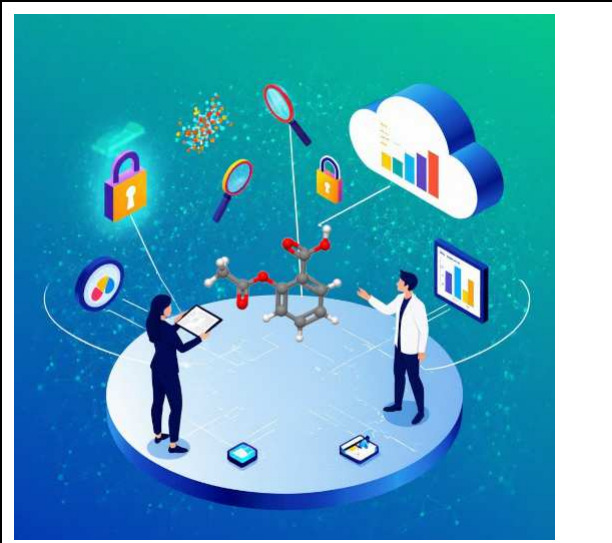

## Introduction

The rapid progress in artificial intelligence (AI), machine learning (ML), and deep learning (DL) has profoundly reshaped the landscape of molecular design and drug discovery.^1,2^ In particular, the emergence of deep generative models (DGMs),^3–12^ reinforcement learning^13^ approaches, and large-scale chemical language models^14^ has enabled the automated creation of vast numbers of molecular structures with promising physical and pharmacological properties. These methods have unlocked new opportunities to explore previously inaccessible regions of chemical space, offering the potential to accelerate early stage drug discovery, advance materials science, and provide new tools for chemical biology.^15^ The paradigm shift from traditional, iterative experimental design toward data-driven molecular generation is now widely recognized as a promising approach of next-generation computational chemistry.

Despite these advances, a persistent challenge lies in the evaluation of molecules produced by AI systems. While generative approaches can create thousands of candidates, in silico, per second, only a fraction of these survive successive layers of automated filtering, which typically eliminate molecules based on chemical validity, basic descriptors, synthetic feasibility, or predicted pharmacological properties. After these computational steps, a substantial number of candidates remain that require expert human evaluation. At this stage, experts such as chemists and pharmacologists closely analyze the two-dimensional and three-dimensional structures. They evaluate these structures for their feasibility, relevance, and potential for improvement, based on their expertise and experience. This manual evaluation is indispensable in the discovery pipeline, since experts often identify structural issues or opportunities for refinement that automated tools cannot capture.

However, this step remains fragmented and inefficient. In both academic and industrial contexts, researchers often evaluate candidate molecules using a mixture of local visualization software, ad-hoc file sharing, and group discussions conducted either synchronously or asynchronously. These practices are time-consuming, prone to inconsistencies, and difficult to scale to the volumes of molecules generated by modern AI models. Furthermore, there is no standardized workflow or agreed set of metrics for this manual stage: different groups prioritize different structural or pharmacological considerations, and the criteria for evaluation are highly context and experience-dependent. For example, in material science, a molecule might be evaluated based on its ability to refract light, which involves analyzing its refractive index and structural properties that influence optical performance. In contrast, in drug design, molecules are often filtered based on their solubility, where researchers assess factors like hydrophilicity and lipophilicity to predict how well a compound will dissolve in bodily fluids, which is crucial for its bioavailability and therapeutic effectiveness. As a result, the collaborative assessment of AI-generated molecules is often hindered by the lack of a shared digital environment tailored to expert-driven inspection.

To address this unmet need, a **web-based, open-source platform**, dubbed MolVE, has been developed specifically to support the manual human evaluation of molecular candidates. The system provides a secure environment for storing, organizing, and visualizing molecules, with particular emphasis on enabling experts to examine structures in both 2D and 3D with common metrics. By relying on containerized deployment, the platform ensures easy installation and scalability across academic and industrial environments. Features such as data set management, interactive visualizations, and user authentication facilitate collaborative workflows, allowing distributed teams of specialists to **asynchronously** curate, annotate, and discuss candidate molecules in a shared online space. Additionally, MolVE offers an Application Programming Interface (API) for seamless integration with programming languages and provides a Python service to run machine learning and deep learning models. These features are especially important as they open the possibility to use MolVE predictions to support the training of deep learning models using Reinforcement Learning,^13,16^ as well as to support human evaluations with AI tools.

It is important to highlight that MolVE’s versatility allows it to be used with any set of molecules, not only those generated with AI models, making it broadly applicable to different research areas such as advanced material science and de novo drug design. In the specific context of drug generation, MolVE aims to expedite and streamline the human evaluation phase, offering a valuable tool for assessing new drug candidates effectively.

In summary, MolVE can be regarded as a tool for accelerating scientific research, providing rapid and effective support for the human-evaluation activity.

## The Web Platform: MolVE

This section outlines the primary features of the platform, focusing on its ML and DL capabilities and implementation specifics, illustrating how the application is built using up-to-date and reliable software technologies.

## Features Highlights

MolVE, whose main components are visible in Figure 1, is meticulously designed to support the expert-driven evaluation of generated molecular candidates by offering a secure, collaborative, and interactive platform. The foundation of the system is its robust user authentication and account management system that ensures the protection of sensitive data and enables accountability. Users access the platform through a login-based system (Figure 1a), which not only secures the environment but also allows administrators to track individual contributions to the evaluation process. There are two types of user accounts: administrators, who have expanded privileges and access to an admin panel, and normal users, who focus solely on evaluating molecules.

**Figure 1:**
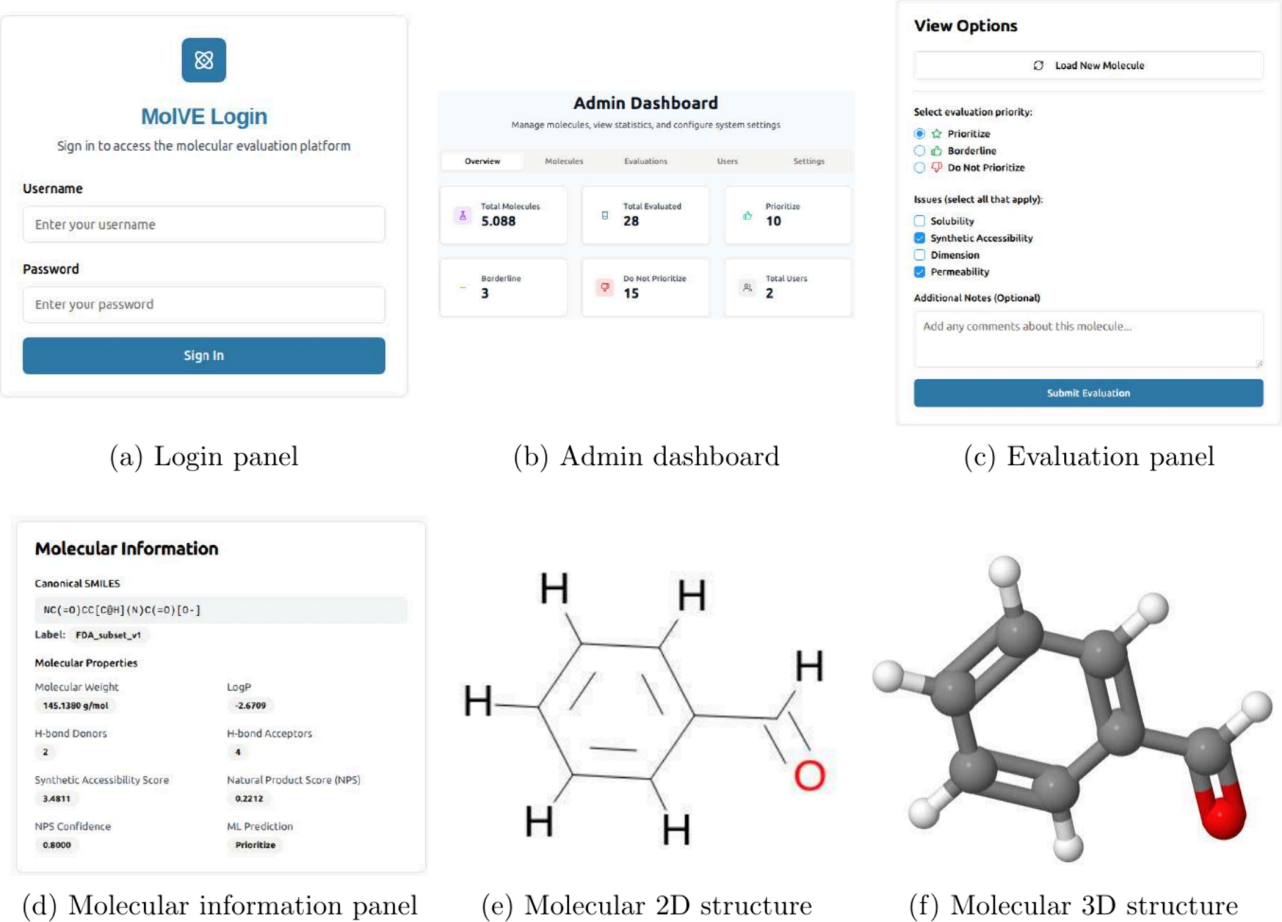
Overview of the main components composing MolVE. (a) The login panel allows users to access the platform securely. (b) The admin dashboard provides an overview of system statistics and user management. (c) The evaluation panel enables users to evaluate molecules using different criteria. (d) The molecular information panel displays detailed properties of a selected molecule. (e) The 2D representation of the molecule. (f) The 3D interactive representation of the molecule.

The admin panel serves as a comprehensive dashboard (Figure 1b), offering a panoramic view of platform activities. It provides administrators with key metrics such as the total number of molecules and an aggregate count of evaluations. This feature is crucial for monitoring the progress and effectiveness of the evaluation process. Additionally, administrators can manage the molecules under evaluation by uploading or removing Spatial Data Files (SDF), i.e., the standard representation used for ligands, and reviewing evaluations submitted by users. This ensures that all evaluations meet quality standards and remain consistent across the board. Furthermore, administrators can manage user accounts, altering access levels or adding and removing users as necessary, and decide on access control policies regarding whether molecules can be viewed without logging into the platform.

A distinctive functionality within the admin panel is the ability to control how molecules are displayed to experts. Admins have the flexibility to choose between three display modes for molecule evaluation, allowing them to customize the process to suit specific research goals or operational requirements:

- *Random Display Mode.* This mode enables experts to assess molecules multiple times, allowing each molecule to receive evaluations from different perspectives. For example, in drug design, a molecule might need to be evaluated by chemists for its synthesis feasibility while pharmacologists concurrently assess its potential efficacy and safety as a therapeutic agent. This is particularly beneficial when aggregating information at a later stage, as it enables the weighting of opinions from various experts. Additionally, experts can re-evaluate the same molecule more than once, which is valuable for analyzing the consistency of their evaluations. For instance, high variance in evaluations by the same expert may indicate randomness in their assessment process. Such analyses, conducted by admins on postevaluation data, may provide insights into the reliability of the evaluations.
- *Unassessed-First Display Mode.* This mode prioritizes molecules that have not yet been evaluated, ensuring a broad coverage of evaluations across a large data set. This is particularly useful when there is a need to quickly gather assessments for a wide range of molecules.
- *Consensus-based Display Mode.* MolVE enables the loading of different molecule sets, each tagged with a label, such as the name of the generating model. Based on this feature of MolVE, this evaluation mode prioritizes showing molecules belonging to more sets. This approach is inspired by the concept of a committee of experts, where each model acts as an individual expert proposing potential molecules. When multiple models independently suggest the same molecule, it indicates a strong consensus and enhanced reliability, similar to a committee reaching an agreement, warranting a higher evaluation priority.

The evaluation page, accessible to both administrators and normal users, is the primary interface for molecular assessment. It presents key molecular properties (Figure 1d) such as the Synthetic Accessibility Score (SAS),^17^ the Natural Product Score (NPS)^18^ along with its prediction confidence, molecular weight, LogP, and hydrogen bond donors and acceptors to inform user evaluations. Additionally, it features predictions from a machine learning model regarding the prioritization of the molecule. The platform employs JSmol^19^ and ChemDoodle^20,21^ for **interactive** 3D (Figure 1f) and 2D (Figure 1e) visualizations, allowing users to manipulate molecular structures by rotating, changing colors, and altering display settings. This interactivity enhances the user’s ability to understand molecular geometry and properties. In addition to these features, the evaluation page provides access to detailed molecular information, including atomic coordinates and SMILES^22–24^ representation, which can also be downloaded as .sdf files for offline analysis or further properties calculation.

The evaluation interface itself supports structured feedback through a qualitative rating scale (Figure 1c): “Prioritize”, “Borderline”, and “Do Not Prioritize”. Additionally, users can specify issues related to molecular structures, “Solubility”, “Synthetic Accessibility”, “Dimension”, and “Permeability”, using checkboxes and provide further insights through textual descriptions. This descriptive scale was chosen to follow the aim of MolVE (i.e., to prioritize or not a molecule) and to accommodate the diverse perspectives and experiences of chemists, who may hold varying opinions on the structural and feasibility aspects of the same molecule. Indeed, experts’ evaluations, such as those from chemists and pharmacologists, may be heavily influenced by their individual backgrounds and expertise, leading to different interpretations and assessments of molecular viability. Thus, for instance, a numerical scale from 0 to 10 introduces challenges in maintaining consistency and meaning, as the variability in experts’ opinions could result in high variance in scores. By providing a descriptive set of granular categories, the platform simplifies the evaluation process, allowing chemists to express their assessments in broader, more easily comparable terms, directly aligned with the ultimate goal of deciding whether to proceed with the molecule to further stages. This approach facilitates clearer communication and consensus-building among evaluators, ensuring that feedback remains meaningful and actionable across different users.

The system is designed with flexibility in mind, allowing admins to download the final evaluations complete with all necessary information, such as the user identity, scores, comments, and evaluation dates. This comprehensive data export is intended to support diverse research scenarios by providing raw data for analysis. Importantly, the platform intentionally refrains from offering any built-in automatic analysis of these results. The absence of specific analytical tools ensures that the platform remains adaptable for various applications, rather than being constrained by predefined analytical frameworks.

## Unique Capabilities

Taken together, all the capabilities of MolVE render it unique in existing cheminformatics platforms. First, it is explicitly centered on qualitative expert prioritization rather than only on visualization or property calculation, providing a role-based, web-based environment for large-scale, structured human assessment of molecules. Second, it offers configurable evaluation modes (Random Display Mode, Unassessed-First Display Mode, and Consensus-based Display Mode), which directly support study designs where different AI models or data sets must be compared under controlled expert evaluation. Third, MolVE tightly integrates a backend Python service for ML and DL models, enabling automatic scoring of uploaded compounds and seamless use within reinforcement learning workflows. Finally, all evaluations (scores, flags, comments, timestamps, and user identities) are exported in a standardized format, so that they can be reused to train or refine AI models, turning expert feedback into machine-readable supervision.

## Machine Learning and Deep Learning Features

MolVE allows the running of Machine Learning (ML) and Deep Learning (DL) models to enhance the evaluation process. Specifically, its current version utilizes a ML model to suggest the prioritization score of a given molecule. This feature offers significant potential, such as enhancing the human evaluation process and enabling the automatic prioritization of molecules within the system. In fact, each molecule uploaded to the platform is automatically assessed, and this predictive functionality is accessible via API, allowing for seamless integration into reinforcement learning^13,16^ models.

The current machine learning model is based on Random Forest, utilizing patterns from a data set where each molecule is represented by Morgan fingerprints.^25^ This data set includes FDA-approved drugs and TumFlow-generated molecules.^3^ It is divided into training and test sets. FDA-approved drugs are marked as “to prioritize,” while TumFlow molecules with high synthetic accessibility scores are “do not prioritize.” To ensure robustness, 5-fold cross-validation is used during training, enhancing generalization by evaluating the model across different data subsets. The integration of this model allows MolVE to effectively integrate AI-generated insights with user evaluations.

It is important to note that, while the current model is trained on a predefined set of molecules, it lays the groundwork for future integration of expert evaluations collected through MolVE into the training process. Inspired by strategies in Natural Language Processing, where DL models are trained to emulate human feedback, a similar approach is envisioned for molecular evaluation. Thus, future work involves enhancing the current model to replicate expert assessments, thereby enabling efficient evaluation of a broader set of molecules, scaling its capabilities beyond prioritization scores to predict structural issues such as synthesizability and permeability.

## Implementation Details

The platform, whose diagram is visible in 2, is a full-stack solution designed to deliver a seamless user experience through a well-structured client-server model. It is divided into four main components: the client-side interface, the backend API, the database, and the Python service API (Figure 2). The entire application is built using free, open-source technologies, enhancing its cost-effectiveness and allowing for extensive customization and scalability.

**Figure 2:**
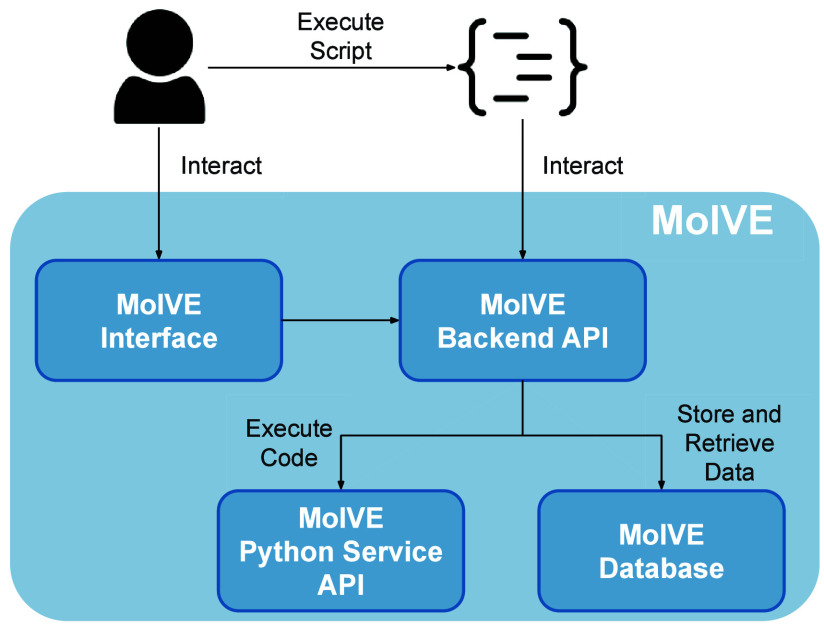
Diagram presenting the main components of MolVE.

On the client side, React^26,27^ is used for its component-based architecture, which promotes the creation of modular and reusable UI components. This is complemented by Tailwind CSS,^28^ which enhances the user interface with a utility-first approach, enabling rapid and responsive styling that seamlessly integrates with React components.

On the server side, Node.js^29,30^ powers the backend with its nonblocking, event-driven architecture, ideal for building scalable network applications. Vite^31^ is chosen as the build tool and development server due to its fast compilation times and efficient hot module replacement, which together significantly enhance the developer experience. Express^32^ is integrated to handle server-side logic and routing, offering a robust framework for managing HTTP requests. Security and session management are addressed with Passport,^33^ used alongside express-session, ensuring user sessions are managed securely and efficiently. Throughout the application, TypeScript^34,35^ is employed, offering static typing that improves code quality by catching errors at compile time.

For database management, PostgreSQL^36,37^ is utilized for its reliability and capability to handle complex queries and transactions. The integration of Drizzle ORM^38^ further simplifies database interactions, providing a type-safe and efficient method for querying and managing database schemas, thereby enhancing both code reliability and developer productivity.

The Python service, built using FastAPI,^39^ is deployed within an independent system that interacts exclusively with the MolVE backend, thereby enhancing security by isolating it from external access.

Containerization is a critical aspect of the deployment strategy, achieved through Docker^40,41^ and Docker Compose.^42,43^ This approach ensures that the application runs consistently across different environments, from development to production, which streamlines the deployment process. Docker encapsulates the application and its dependencies into a single, portable container, while Docker Compose automates the deployment of the database and other services, providing a quick, ready-to-use, and dependable setup.

## Related Works

To the best of current knowledge, there is no web-based tool offering the same combination of features as MolVE. The closest related system is Metis,^44^ a Python-based graphical interface that lets chemists provide feedback on de novo generated molecules, including preferred substructures and project constraints. Integrated with REINVENT,^45^ Metis incorporates this feedback into the reward model, supporting iterative, closed-loop design. MolVE is complementary: whereas Metis is a desktop-style Python application designed for chemists or small groups within a specific de novo design loop, MolVE is a general-purpose, web-based platform for large-scale, distributed, and asynchronous expert evaluation of molecular sets. Key differences include:

- *Deployment and accessibility.* MolVE is a browser-accessible, Dockerized web application backed by PostgreSQL, requiring no local installation. Metis relies on a local Python stack and is not currently web-hostable.
- *Collaboration and scale.* MolVE supports role-based user management, authentication, and display modes (random, etc.), enabling coordinated evaluation of large molecular collections by distributed teams. Metis is optimized for interactive, project-centric feedback on a particular de novo design run.
- *Integration and focus.* MolVE exposes generic REST APIs and a FastAPI-based Python service for arbitrary ML/DL models, emphasizing standardized qualitative prioritization (“Prioritize”, etc.) with structured issue flags and integrated predictions across heterogeneous molecule sets. Metis is tightly coupled to REINVENT and focuses on detailed, often substructure-level feedback to build reward models and refine a specific design campaign.

In summary, both Metis and MolVE support human-in-the-loop generative chemistry but target different scenarios: Metis as a Python-native interface within a specific de novo design framework, and MolVE as a web-based, multiuser platform for scalable, model-agnostic molecular evaluation and prioritization.

Beyond Metis, a variety of cheminformatics tools provide molecular visualization, property calculation, or workflow management. These include standalone applications and libraries, e.g., RDKit-based^46^ viewers and scripting environments, DataWarrior,^47^ as well as web-based viewers primarily focused on 2D/3D rendering and basic descriptor computation. However, such tools are generally oriented toward single-user analysis or batch property calculation and do not offer a role-based, browser-accessible environment for structured, large-scale expert prioritization of molecular sets. In contrast, MolVE is explicitly designed to support distributed, multiuser evaluation with configurable display modes and standardized qualitative feedback, tightly integrating human judgments with machine learning models in a single, centralized platform.

## Case Study: Evaluation of TumFlow Generated Molecules

To illustrate how MolVE integrates with external machine learning models and supports expert assessment, we conducted a small case study using molecules generated by TumFlow.^3^ A set of TumFlow-generated structures was imported into MolVE via its REST API, automatically parsed from SDF format, and stored together with metadata identifying TumFlow as the source model. For each imported molecule, MolVE’s backend invoked the integrated machine learning (ML) model to produce a preliminary prioritization suggestion. These predictions were displayed alongside computed molecular properties (e.g., SAS, NPS, molecular weight, LogP) and interactive 2D/3D visualizations on the evaluation page. A small group of medicinal chemists then used MolVE to review a subset of these TumFlow-generated molecules. Experts accessed the platform through a standard web browser, without installing any additional software, and evaluated molecules using the “Unassessed-First” display mode to ensure broad coverage of the set. For each structure, they assigned a qualitative label (“Prioritize”, “Borderline”, or “Do Not Prioritize”), optionally flagged issues such as solubility, synthetic accessibility, size, or permeability, and recorded free-text comments when needed.

Even at this limited scale, the case study highlighted distinctive aspects of MolVE:

- *Model-agnostic integration.* TumFlow-generated molecules were ingested through API, demonstrating that MolVE can serve as a generic, web-based front end for diverse generative models and external pipelines.
- *Unified human–AI assessment.* ML-based prioritization suggestions and cheminformatics descriptors were presented in the same interface as expert ratings and comments, enabling chemists to quickly identify and focus on molecules where human judgment and model predictions disagreed.
- *Distributed, role-based evaluation.* All evaluations, including user identities, timestamps, qualitative labels, issue flags, and comments, were recorded in a centralized PostgreSQL database. This allowed administrators to monitor coverage of the TumFlow set, export standardized evaluation data for downstream analysis, and potentially reuse these annotations to refine future ML models.

Rather than aiming for exhaustive coverage of all TumFlow-generated molecules, this case study was designed to demonstrate MolVE’s practical deployment in a realistic setting: connecting an external generative model, automatically scoring its outputs, and enabling expert review in a structured, reproducible manner within a single web-based platform.

## Conclusions and Future Works

MolVE offers a comprehensive solution to the growing challenge of human-evaluating molecular candidates in advanced material science and drug discovery. This secure, open-source, web-based platform is designed for asynchronous, distributed, and collaborative evaluation by experts. It features interactive 2D and 3D visualizations, API integration, ML predictions, and a structured qualitative feedback system, streamlining expert assessments while accommodating diverse evaluation criteria. Its full-stack, containerized architecture ensures scalability and ease of deployment in both academic and industrial contexts. By supporting rapid and effective human evaluation, MolVE accelerates scientific research, facilitating more efficient and reproducible decision-making in the early stages.

Future work will focus on utilizing the platform to collect a comprehensive data set for training a machine learning model that can enhance molecule prioritization predictions beyond the capabilities of the current model. With this new data set, the model could also be trained to identify potential structural issues, a capability not present in the existing model. This advancement could significantly improve decision-making by providing rapid, expert-like evaluations of molecules, thereby streamlining the early phases of drug discovery. Additionally, future efforts will aim to integrate machine learning and deep learning models to evaluate various properties, such as toxicity. Deep learning models will also be trained to generate new molecules, with a specific focus on creating high-priority candidates using reinforcement learning facilitated by MolVE. Finally, future work will concentrate on incorporating collaborative features, advanced analytics, and ensuring interoperability with other computational pipelines to further enhance the evaluation process.

## Data Availability

The ready-to-use code, containerized with Docker, is available on GitHub (https://github.com/drigoni/MolVE).
